# 17.4 POSSIBLE MECHANISMS INVOLVED IN THE ANTIPSYCHOTIC EFFECTS OF CANNABIDIOL (CBD)

**DOI:** 10.1093/schbul/sby014.068

**Published:** 2018-04-01

**Authors:** Jose Alexandre Crippa, Antonio Waldo Zuardi, Francisco Silveira Guimaraes

**Affiliations:** University of Sao Paulo

## Abstract

**Background:**

Laboratory rodents and human studies have demonstrated that cannabidiol (CBD) presents antipsychotic effects. Several mechanisms of action have been associated to CBD effects. Most of the studies that have investigated these mechanisms have been performed in vitro and their relevance for the in vivo effects of this drug is still uncertain.

**Methods:**

In spite of its low affinity for CB1 receptor (CB1R), CBD is capable of antagonizing CB1R agonists at reasonable low concentrations. CBD can also inhibit anandamide uptake and metabolism, enhancing endocannabinoid tonus. It is also possible to attribute the antipsychotic action of CBD to an antagonism of CB1R. This suggestion was reinforced by the observation that patients in the initial prodromal states of psychosis and patients with schizophrenia had higher levels of anandamide in CSF than health controls. Moreover, there would be a decrease in glutamatergic inputs from this area to the prefrontal cortex, impairing locomotor activity and working memory and endocannabinoids could regulate this system. CBD could facilitate endocannabinoids “on demand” synthesis in post-synaptic neurons, acting pre-synaptic terminals and negatively regulating the release of neurotransmitters, particularly GABA and glutamate.

**Results:**

Also, an increase in anandamide levels induced by CBD could attenuate GABA release from ventral pallidum neurons, restoring the normal function of this system in psychotic patients. CBD can also increase adult neurogenesis in mice and that this effect has been shown to be dependent on the CB1 receptors. In addition to the mechanisms discussed above, CBD can produce several other effects that could also be involved to be responsible for its antipsychotic properties, including interaction with 5HT1A and TRPV1 receptors and anti-inflammatory/neuroprotective effects. CBD can act as a serotonin 1A receptor (5HT1A) agonist, although the role of 5-HT1A-mediated neurotransmission in schizophrenia is unclear, aripiprazole, an atypical antipsychotic, acts as a partial agonist at this receptor, an effect that could, together with its actions on D2 and 5-HT2A receptors, contribute to therapeutic effects of this drug. CBD can also activate Transient Receptor Vanilloid-1 (TRPV1) receptors that are expressed in several brain areas related to psychosis such as the prefrontal cortex, amygdala and hippocampus. Since CBD is also a potent anti-inflammatory, antioxidant and neuroprotective compound, it is possible that these effects are involved in its antipsychotic action.

**Discussion:**

Since CBD is also a potent anti-inflammatory, antioxidant and neuroprotective compound, it is possible that these effects are involved in its antipsychotic action.

